# How age and sex interaction is associated with immune aging with implications for vaccines and age-related autoimmunity

**DOI:** 10.1038/s41514-026-00491-6

**Published:** 2026-08-22

**Authors:** Reza Gheitasi, Mahnaz Rezaei, Parvaneh Nafisi Fard, Daniela Roel, Sabine Baumgart, Diana Dudziak, Norman Rose, Simon M. Petzinna, Thomas Kamradt, Carsten Watzl, Mathias W. Pletz, Valentin S. Schäfer

**Affiliations:** 1 Clinic of Internal Medicine III, Oncology, Hematology, Rheumatology and Clinical Immunology, University Hospital Bonn, Bonn, Germany; 2 Immunodeficiency Diseases Research Center, Isfahan University of Medical Sciences, Isfahan, Iran; 3 Division of Molecular Cell Biology, Institute of Theoretical Medicine, Faculty of Medicine, University of Augsburg, Augsburg, Germany; 4 Institute of Infectious Diseases and Infection Control, Jena University Hospital/Friedrich Schiller University, Jena, Germany; 5 Institute of Immunology, Jena University Hospital/Friedrich Schiller University, Jena, Germany; 6 Department of Immunology, Leibniz Research Centre for Working Environment and Human Factors at the Technical University Dortmund (IfADo), Dortmund, Germany

**Keywords:** Diseases, Immunology

## Abstract

Immunosenescence is not a uniform decline but a trajectory shaped jointly by age and sex. Using continuous high-dimensional immunophenotyping, we show that aging T cells accumulate a senescent signature of CD28 loss and CD57 gain, and that males reach an inverted CD4:CD8 ratio earlier and more severely than females. We argue that age-by-sex stratification should guide vaccination and geroprotective strategies in an aging population.

## Introduction

The global demographic shift toward an aging population has intensified focus on the physiological decline that underpins age-related diseases. Central to this decline is immunosenescence, a complex and multifaceted remodeling of the immune system that erodes its ability to mount effective responses against novel pathogens, control malignancy, and maintain self-tolerance^1^. While immunosenescence is universally recognized as a hallmark of aging, its traditional characterization as a simple, uniform decay fails to capture the profound heterogeneity and clinical nuances of the process^2^. A more sophisticated understanding is required to navigate the growing burden of age-related illnesses, from diminished vaccine efficacy to mount effective responses against novel pathogens, control malignancy, and maintain self-tolerance. This perspective argues for a translated clinical framework that considers immunosenescence not as a uniform, linear decline, but as a heterogeneous trajectory fundamentally shaped by the critical interplay of age, sex, and cumulative antigenic stress. Recent reviews confirm that immune aging follows distinct trajectories between the sexes, with a majority of studies now documenting sex-specific differences that influence both innate and adaptive immune responses^3–5^. The immunological landscape of an aging individual is a product of life-long cellular adaptations and compromises, which manifest with distinct patterns in males and females^3^. We hypothesize that not accounting for these sex-dimorphic trajectories limits our ability to develop personalized and effective interventions. In this perspective, we first outline the age- and sex-related changes in key immune cell populations, including dendritic cells and the adaptive T and B lymphocytes. A structured literature search identified consistent evidence that aging T cells lose CD28 expression, a change linked to reduced proliferative capacity and impaired vaccine responses^6–8^. We will then explore how these cellular-level alterations give rise to the state of chronic, low-grade inflammation known as inflammaging and drive the progressive breakdown of immune tolerance. Building on this foundation, we will connect these cellular deficits to two major clinical consequences: the attenuated efficacy of vaccines in older adults and the pathogenesis of age-related autoimmune diseases, such as giant cell arteritis (GCA) a granulomatous systemic large vessel vasculitis^9–11^. We will conclude by proposing a new paradigm for therapeutic and prophylactic strategies that explicitly incorporates age- and sex-specific insights, charting a course toward more precise and effective immune health management for an aging population. Aging induces complex remodeling across the innate and adaptive immune systems, a process that defies a uniform description^12^. Rather than a simple decline, each cell lineage experiences distinct alterations that are shaped by both age and sex, leading to divergent immune trajectories.

## Dendritic cells phenotypical changes in aging

Dendritic cells (DCs), the antigen-presenting cells that initiate immune responses and maintain tolerance, are central to age-related remodeling. While DC numbers in the blood may remain stable in some cohorts, their function deteriorates significantly^13–15^. The numerical changes are often conflicting across studies, with some finding a significant age-related decline in plasmacytoid DCs (pDCs) after age 50, but not myeloid DCs (mDCs)^16–18^, while others report a progressive decrease in mDCs with age^19^. These discrepancies may arise from differences in methodology or, importantly, the specific populations studied, highlighting that geographic, genetic, and environmental backgrounds (such as endemic pathogen exposure and socio-economic determinants) act as critical covariates that further modulate these immune aging trajectories. Regardless of their numerical stability, the functional decline of aged DCs in older adults is reported, in the way that mDCs produce less pro-inflammatory cytokines such as tumor necrosis factor (TNF), interleukin(IL)-6, and IL-12 upon stimulation^20^. Similarly, pDCs, the primary producers of type I interferons, show a blunted antiviral program, secreting significantly less interferon alpha (IFN-α) in response to toll-like receptor (TLR)7 and TLR9 ligands. This defect is linked to impaired activation of the interferon regulatory factor (IRF)-7 transcription factor and results in a weaker initial antiviral defense^20–22^. Overall, aged DCs exist in a state of chronic activation at baseline yet are paradoxically less able to mount a robust, coordinated response to new pathogens^23^. These age-related changes are critically modulated by sex hormones. Thus, estrogen signaling through estrogen receptor alpha (ER-α) can enhance TLR-mediated responses in human pDCs, leading to increased IFN-α production^24–26^. This enhanced female responsiveness is further influenced by the X-chromosome complement, as incomplete X-chromosome inactivation can result in a higher TLR7 gene dosage in females, contributing to greater IFN-α production in a cell-autonomous manner^27^. This effect is observed in both in vivo and in vitro studies and helps explain why females generally mount stronger viral defenses but are also more susceptible to IFN-α-driven autoimmune diseases like systemic lupus erythematosus (SLE)^28^. While some studies find modest sex differences in circulating DC counts, with some evidence of lower total DCs and mDCs in younger females that diminish post-menopause, the functional divergence mediated by hormonal milieu is clear: female DCs tend to have a more potent cytokine response^16,29^.

## Macrophages phenotype changes during immunoaging

Macrophages, derived from circulating monocytes, serve as a frontline defense and can be impacted by aging, too. Although the absolute number of monocytes in the blood tends to increase with age, often driven by a pro-inflammatory milieu^30^, the functionality of the macrophage lineage is compromised. Aged macrophages exist in a state of constitutive pro-inflammatory activation, secreting elevated basal levels of TNF-α and IL-6^31^. Paradoxically, despite this heightened state of readiness, these cells respond less vigorously to acute stimuli. Studies in both humans and mice reveal age-associated declines in TLR activation and subsequent cytokine production, a defect linked to reduced receptor expression and altered signaling pathways^24^. Furthermore, the phagocytic and microbicidal activities of aged macrophages are often compromised, leading to delayed pathogen clearance. Phagocytosis of opsonized E. coli is reportedly reduced in elderly individuals^32^, and there is a diminished capacity to produce reactive oxygen species and nitric oxide (NO)^33^. This complex dysregulation, where an elevated pro-inflammatory baseline coexists with an impaired acute response, exemplifies how aging macrophages contribute to chronic inflammation rather than an efficient, protective immune response. The aged environment itself, with its increased senescent stromal cells and high cytokine levels, further drives macrophages toward this maladaptive, pro-inflammatory state^34^. Sex differences in macrophage aging are less well-defined, though hormonal influences likely modulate their function^34^.

## T cells phenotype shift from naive to senescent

**Fig. 1:**
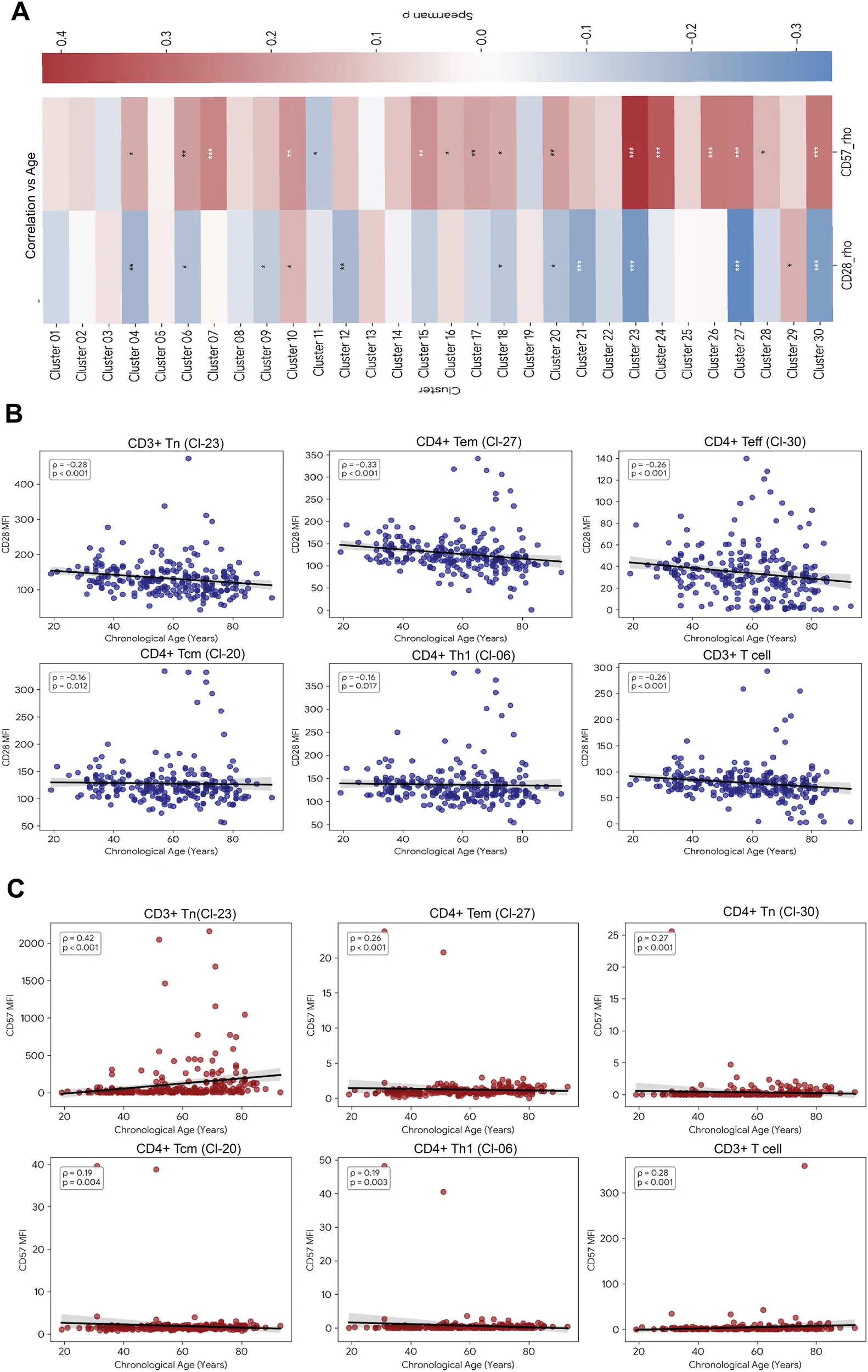
Signature of immunosenescence defined by concurrent CD28 loss and CD57 accumulation across distinct T cell subpopulations. **A** Global Spearman correlation heatmap illustrates the relationship between continuous chronological age and the expression of CD28 and CD57 across 30 high-dimensional T cell clusters. Asterisks denote statistical significance (*p < 0.05, **p < 0.01, ***p < 0.001). **B**, **C** Scatter plots detailing the significant, continuous age-dependent decline of CD28 (**B**) mean fluorescence intensity (MFI) in the identified dual-signature populations: Tn (Cluster 23), CD4+ Tem (Cluster 27), CD4+ Teff (Cluster 30), CD4+ Tcm (Cluster 20), CD4 + Th1 (Cluster 06), and total CD3 + T cells. Solid black lines represent linear regression fits, with shaded gray regions indicating 95% confidence intervals. Non-parametric Spearman’s rank correlation coefficients *(*rho) and associated *p*-values are displayed. **C** Corresponding scatter plots demonstrating the significant, continuous age-dependent increase of CD57 MFI in the identical dual-signature populations.

**Fig. 2:**
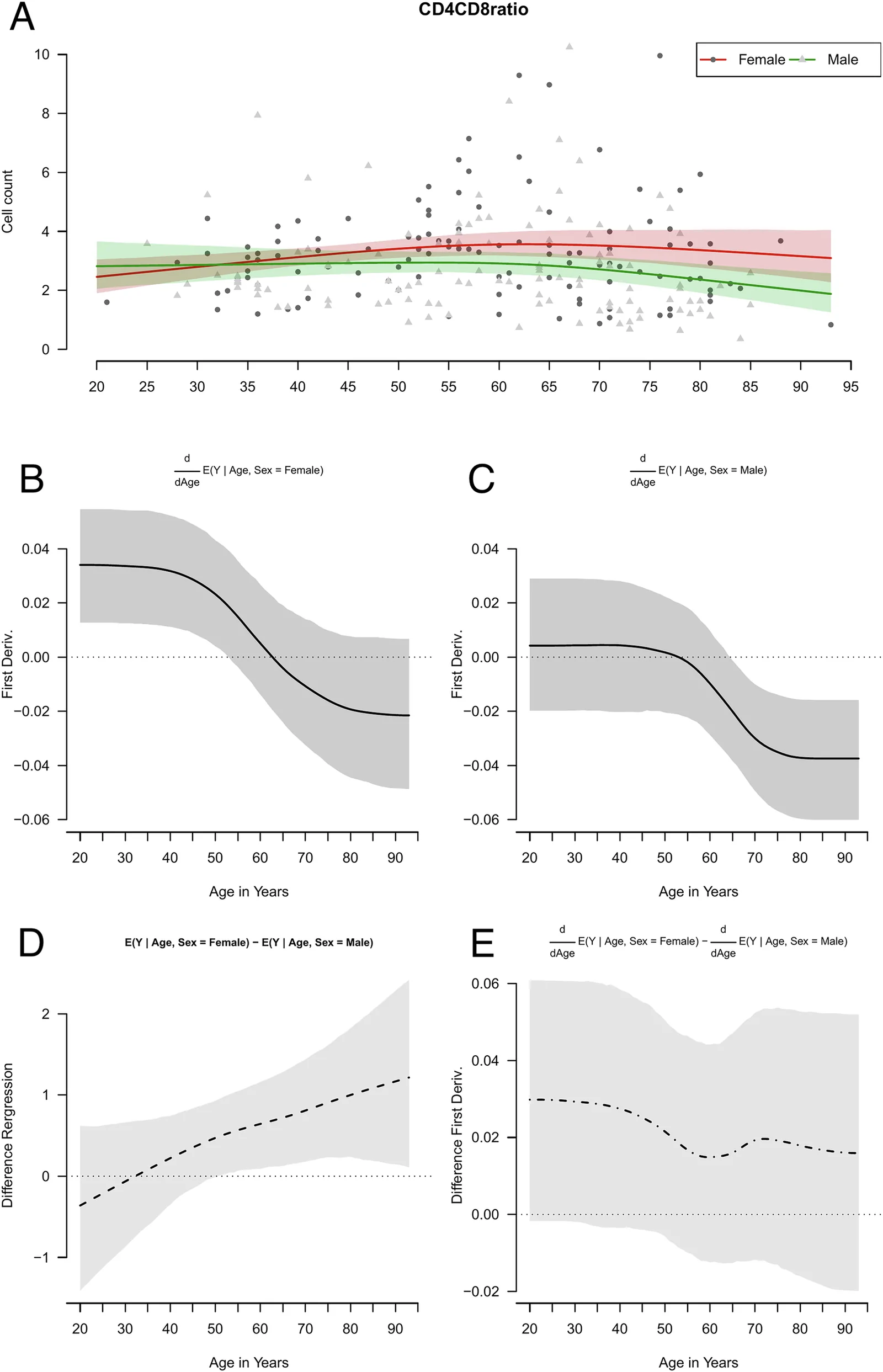
Absolute CD4:CD8 ratio trajectories and derivative analyses by sex across the lifespan. This figure illustrates the age-related changes in the CD4:CD8 T cell ratio, utilizing spline regression models to highlight distinct patterns between sexes. **A** The primary graph presents the absolute CD4:CD8 ratio for male (green) and female (red) individuals across a continuous age range. Each point represents an individual participant, with solid lines indicating the fitted spline regression and shaded areas representing the 95% confidence intervals. The first derivative of the expected CD4:CD8 ratio with respect to age is shown for females (**B**) and males (**C**), illustrating the instantaneous rate of change in the ratio at any given age. **D** Differences of the sex-specific regression curves at each age with the 95% confidence band. Significant sex differences at a given age are inferred where the 95% confidence band of the curve difference excludes zero. Hence, a significantly higher CD4:CD8 ratio in females is found from approximately 48 years of age onwards. The continuous increase of the mean differences across the lifespan is due to the divergent trajectories, specifically the progressive decline of the CD4:CD8 ratio in males compared to a relatively stable ratio in females. **E** Difference of the slopes of the sex-specific regression curves at each age with the 95% confidence band. In general, differences in the slopes of the sex-specific regression curves indicate the interaction between age and sex. Statistically significant differences of the slopes, and therefore a significant interaction effect, are indicated by 95% confidence intervals that do not overlap with zero. For the CD4:CD8 ratio, the 95% confidence intervals overlap with zero across the investigated age range. This indicates that while the absolute ratios diverge significantly in later life (as seen in panel D), the instantaneous difference in the rate of change (slope) between sexes at any single specific age point does not reach statistical significance.

The most profound age-related changes occur in the T cell compartment, a process initiated by the progressive involution of the thymus from puberty onward^35^. This process sharply curtails the production of new naive T cells, leading to a drastic shrinkage of the naive T cell pool^35,36^. As a compensatory mechanism, existing T cells undergo homeostatic proliferation, a process that, over time, drives them toward replicative senescence^37^. The result is a major shift in T cell composition. The naive T cell pool declines while highly differentiated memory and senescent T cells accumulate. Terminally differentiated effector memory CD8 + T cells (TEMRA), which are characterized by the loss of the co-stimulatory molecule CD28, become more prevalent with age^16^. This process has been linked to chronic viral infections like cytomegalovirus (CMV), which accelerate CD28 loss^37^. Our continuous trajectory analysis revealed a dual-signature of immunosenescence, defined not only by the consistent loss of CD28, which is linked to impaired proliferation and reduced responsiveness^38^ but also by the simultaneous accumulation of the terminal differentiation marker CD57 (Fig. 1A). This concurrent CD28 loss and CD57 gain was significantly pronounced across distinct T cell subpopulations over the lifespan, including CD4+ effector T cells (Teff), CD4+ effector memory T cells (CD4+ Tem), CD4+ central memory T cells (CD4+ Tcm), CD4 + Th1 cells, and the total CD3 + T cell pool (Fig. 1B). Interestingly, among these subsets, we identified that even the naive T cell (Tn) phenotype exhibited this senescent trajectory, progressively losing CD28 and acquiring CD57 across the lifespan (Fig. 1B). A newly recognized subset called virtual memory T cells, also expands with age. Although initially functional, these cells gradually lose their TCR-driven proliferative capacity and adopt a senescent-like phenotype, further contributing to the repertoire rigidity^38^. Crucially, these T cell aging trajectories are sex-dependent. Our previous study confirmed that women often maintain better-preserved naive T cell populations and higher T-cell receptor excision circle (TREC) levels, suggesting a slower rate of immunosenescence compared to men^39^. Men tend to exhibit an inverted CD4:CD8 ratio earlier or more severely (Fig. 2)^16^. Furthermore, it has shown that age-associated increases in N-glycan branching, which suppresses T cell activation, are more pronounced in female naive CD4 T cells^40^. This finding points to sex-dimorphic molecular mechanisms that influence T cell function, with potential links to IL-7 signaling and metabolic changes. Collectively, these differences suggest that men exhaust their naive T cell reserves more rapidly, while women may face stronger age-related signaling blocks on activation, creating divergent challenges for immune health. As a result, studies that compile different age-dependent immunological markers into a single composite score find that the immune system of females is about a decade younger than the immune system of males of the same chronological age^41,42^.

The cellular and functional reprogramming of the immune system with age is not an academic phenomenon; it has profound clinical ramifications that impact an individual susceptibility to disease and their responsiveness to therapeutic interventions. The breakdown of immune surveillance and the shift toward a pro-inflammatory state compromise the body’s ability to defend against pathogens, control malignancy, and maintain self-tolerance. The consequences are particularly evident in the diminished efficacy of vaccines and the increased prevalence of age-related autoimmune diseases.

## Attenuated vaccine efficacy in the elderly

Vaccination is a cornerstone of preventative medicine, yet its intrinsic effectiveness wanes significantly with age, a direct consequence of immunosenescence^11^. While frequent mutations in pathogens such as Influenza and SARS-CoV-2 necessitate seasonal or updated formulations, older adults independently mount weaker, delayed, and less durable antibody and T cell responses to these vaccines compared to younger individuals^43,44^. This reduced responsiveness is linked to the age related decline in naïve T cells and the loss of CD28 expression, which limits T cell proliferation and cytokine production^45,46^.

We intend to highlight that while age related immune decline is a general trend, vaccine responses vary significantly depending on the vaccine platform and the host’s sex. For instance, studies on influenza vaccines indicate that females may exhibit stronger initial antibody responses but experience a faster subsequent decline, whereas males may have a more sustained but overall less robust initial response^23^. SARS-CoV-2 Vaccine in males, in particular, may require additional doses or more frequent booster shots to achieve and maintain adequate protection^44^. In contrast to influenza and SARS-CoV-2, pneumococcal conjugate vaccines (PCV) have shown a generally well-preserved T-cell-dependent response in older adults, whereas the T-cell-independent responses elicited by pneumococcal polysaccharide vaccines (PPV) are typically less robust^47^. This divergence underscores the importance of the vaccine platform in modulating age-related immune decline. As our previous study has suggested, this may be due to a more robust repertoire of mobile naive B cells^16^ indicating that not all vaccines are equally affected by immunosenescence.

These data suggest that vaccine strategies need to evolve to counter specific age- and sex-related immune deficits. The impairment of vaccine efficacy is also linked to the disruption of the germinal center reaction, where aged T follicular helper (TFH) cells mislocalize, resulting in a less effective B cell response and the production of lower-affinity antibodies^48–50^. Promisingly, advanced vaccine platforms, such as a self-amplifying mRNA (SAM) SARS-CoV-2 vaccine, have shown they can partially overcome these limitations by eliciting robust and durable T cell responses in older adults^43,51^.

## Age-related autoimmunity: the paradox of hyperinflammation

Paradoxically, as the immune system capacity to fight external threats declines, its tendency to attack self-tissue increases, leading to a higher incidence of autoimmune diseases in the elderly. This is a direct consequence of a breakdown in immune tolerance. A prime example is GCA, a vasculitis that occurs exclusively in individuals over the age of 50. The pathogenesis of GCA illustrates how aged immunity fails to maintain self-tolerance. In the vessel wall, arterial DCs that normally reside in a quiescent, tolerogenic state become pathologically activated. These activated DCs spontaneously mature, present self-antigens with full costimulation, and unleash a T cell-mediated attack on the artery^15^. This age-related DC dysfunction, coupled with the chronic pro-inflammatory state of inflammaging, provides a fertile ground for autoimmunity^52^. Another case is bullous pemphigoid (BP), an autoimmune blistering skin disease that predominantly affects the elderly. The BP is driven by a Th2-skewed immune response where autoreactive T cells produce IL-4 and IL-13, prompting B cells to produce autoantibodies against skin proteins. The age-related erosion of T cell tolerance and the systemic shift toward a type-2 inflammatory milieu set the stage for BP^17^. In both cases, immunosenescence creates a perfect storm: impaired clearance of immune complexes alongside a dysregulated, inflammatory environment that both initiates and sustains autoimmune tissue damage. This dual liability poor responses to external threats coupled with heightened internal reactivity underscores the urgent need to understand and intervene in the processes of immunosenescence.

The heterogeneous nature of immunosenescence demands a shift from one-size-fits-all guidelines to personalized strategies that account for sex and individual immune state. We propose a multifaceted approach that leverages our growing understanding of age- and sex-specific immune aging to optimize prevention and therapy.

## Reprogramming vaccination guidelines

Current vaccination schedules are largely based on chronological age thresholds, but this ignores the vast heterogeneity in immune aging. Instead, we advocate for integrating both age and sex into vaccine strategies. Our previous research and others confirm that immune aging unfolds along sex-specific trajectories. We found that key immune cell populations, including memory B cells, naive CD8 + T cells, central memory CD8 + T cells, and MAIT/NKT cells, are better preserved in females with age compared to males^16^. This suggests that the immunological baseline for vaccine responses differs fundamentally between the sexes. Given these distinct immune signatures, vaccine formulations and schedules could be tailored accordingly. For example, vaccines for older men could emphasize strong CD8 + T cell stimulatory signals to compensate for their more rapid decline in this population. Conversely, vaccines for older women might be optimized to leverage their better-preserved memory B cell and T cell composition. We also argue for moving toward an immune risk phenotype to guide dose and booster frequency. Variables such as CMV serostatus, inflammatory markers, and the naive:memory T cell ratio could be used to identify who needs higher antigen loads or more frequent boosters. We also found that comorbidities, such as those captured by the Charlson Comorbidity Index, are associated with cellular hypo-responsiveness to vaccines, underscoring the need to factor in overall health status alongside age and sex. These data collectively argue that vaccination should be a precision intervention, calibrated to each individual immune baseline rather than just their birthdate. The findings on age-related autoimmunity, such as GCA, also suggest that guidelines for treatment should consider the root causes in immunosenescence, rather than simply suppressing symptoms.

## Innovative therapeutic and research strategies

Beyond prophylactic guidelines, targeted interventions must address the fundamental molecular drivers of immunosenescence. Thymic rejuvenation to restore naïve T cell output remains a primary target^16^. Strategies such as sex steroid ablation via luteinizing hormone-releasing hormone (LHRH) antagonists demonstrate efficacy in upregulating thymic IL-7 and delta-like canonical Notch ligand 4 (DLL4) expression. Concurrently, senolytic agents targeting p16-positive cells offer a mechanism to clear the dysfunctional, terminally differentiated T cells that propagate chronic inflammation^53^. Epigenetic and metabolic modulators, including mammalian target of rapamycin (mTOR) inhibitors and histone deacetylase (HDAC) inhibitors targeting the Menin-Bach2 axis, show potential in restoring T cell stemness^54,55^. Moreover, neutralizing the tonic interferon secretion driven by retrotransposon element insertion presents a novel therapeutic avenue to mitigate inflammaging^56^. To translate these findings, future research paradigms must actively investigate age × sex interactions using systems immunology. Large scale longitudinal cohorts must systematically profile participants using standardized, high dimensional immunophenotyping coupled with proteomics and bulk RNA sequencing, ensuring that population-specific genetic architectures and environmental exposures are integrated as core covariates alongside age and sex. The application of machine learning algorithms to these multiomics datasets will facilitate the derivation of robust, sex-specific biomarkers of immune aging. Armed with these high-resolution profiles, subsequent clinical trials can stratify participants by biological immune health rather than chronological age, ensuring the rigorous evaluation of personalized interventions.

## Discussion

Immunosenescence is a central driver of disease in our aging population, but it is not a monolithic decline. The aging immune system is reconfigured in a complex, sex-dependent way: antigen-presenting cells lose viral tolerance and hyper-activate inflammaging, macrophages become chronically pro-inflammatory yet less effective, and T cells shift from diverse naive pools to rigid, senescent phenotypes. Crucially, these changes unfold along different trajectories in men and women, with females retaining a better-preserved repertoire of memory B cells, naïve CD8 + T cells, central memory CD8 + T cells, and MAIT/NKT cells compared to males. This nuanced view demands that we abandon age-only metrics in clinical practice. Instead, we should implement personalized, age-and-sex aware strategies. For example, vaccine regimens can be optimized by sex and by individual immune status, assessed via biomarkers like CMV serostatus or T cell subset ratios. Likewise, treating age-related autoimmunity should target the cellular drivers of immunosenescence. Thymic rejuvenation, senolytics to clear dysfunctional cells, metabolic modulators, and epigenetic agents are all avenues that can restore immune competence rather than merely damp inflammation.

Realizing this vision requires concerted effort: defining precise biomarkers of immune age, including sex as a core variable in studies, and conducting longitudinal trials that test these personalized approaches. By integrating the dynamic biology of immunosenescence into medical guidelines, we can shift from reacting to decline toward proactively preserving immune health in later life. This precision immunology paradigm offers a roadmap to not only extend lifespan, but to ensure those extra years are healthier, with better vaccine protection and fewer autoimmune diseases. While the evidence for sex-dimorphic immunosenescence is compelling, several limitations within the current field must be acknowledged. First, much of the data driving these insights, including our own high-dimensional immunophenotyping, relies on cross sectional cohort studies. While these studies reveal distinct age-related patterns, true longitudinal tracking of individual immune trajectories over decades is required to definitively separate aging effects from generational or environmental confounders. Second, while transcriptomic and phenotypic differences (such as CD28 loss or altered CD4:CD8 ratios) are well-documented, the precise mechanistic links between these cellular markers and clinical outcomes, such as the exact threshold of CD28 + T cells required for a protective vaccine response in a 70 years old female versus male, remain incompletely defined. Third, the age × sex interaction we describe appears to be compartment specific rather than a global feature of immune aging: in the same cohort, the age-associated decline of CXCR5 and accumulation of CD57 across B cell subsets showed no corresponding sex difference after correction for multiple testing, indicating that this dimorphism is a T cell phenomenon. Finally, extrapolating from peripheral blood profiles to tissue resident immune function carries inherent limitations. Future studies must prioritize longitudinal designs, integrate multiomics tissue analyses, and systematically evaluate age × sex interactions to translate these observational perspectives into actionable, personalized clinical guidelines.

## Data Availability

The datasets generated and analyzed during the current study are not publicly available because they contain participant-level immunophenotyping and clinical metadata from the CoNAN cohort that could compromise individual privacy and are governed by the cohort's ethics approval and data-protection terms, but are available from the corresponding author (Reza Gheitasi, Reza.Gheitasi@ukbonn.de) on reasonable request.
